# Coordinated control of acceleration slip regulation and active front steering for in-wheel motor driven electric vehicle based on dynamic slip ratio constraint

**DOI:** 10.1371/journal.pone.0334519

**Published:** 2025-10-23

**Authors:** Zhigang Zhou, Wei Shen, Fang Xu, Ruili Yang

**Affiliations:** 1 College of Vehicle and Traffic Engineering, Henan University of Science and Technology, Luoyang, China; 2 College of Mechanical and Electrical Engineering, Henan University of Science and Technology, Luoyang, China; National University of Singapore, SINGAPORE

## Abstract

In-wheel motor driven electric vehicles are prone to issues such as wheel spin, vehicle sideslip, fishtailing, and steering instability when traveling on compacted snow surfaces. While traditional optimal slip ratio tracking aims to maximize longitudinal force, it significantly compromises lateral force reserves. Moreover, most existing longitudinal-lateral coordination strategies fail to actively constrain the slip ratio, unable to prevent the deterioration of road adhesion conditions. To address these challenges, this study proposes a coordinated control strategy integrating acceleration slip regulation (ASR) and active front steering (AFS) under dynamic slip ratio constraints. To maximize the preservation of tire lateral force margin for enhanced anti-sideslip capability, a dynamic slip ratio constraint control method is proposed. With the objectives of dynamically minimizing wheel slip ratio and maintaining driving stability, the non-dominated sorting genetic algorithm-II (NSGA-II) is employed to optimally distribute the total driving torque. Furthermore, to counteract undesired yaw moments caused by uneven road friction coefficients or torque distribution, an active front steering (AFS) compensation control strategy based on sliding mode control (SMC) is designed to track the ideal yaw rate and sideslip angle, thereby achieving efficient coordination between acceleration slip regulation and yaw stability control. Co-simulations under various conditions are conducted using the Matlab/Simulink-CarSim platform. The results demonstrate that the proposed strategy effectively suppresses wheel spin, reduces lateral path tracking errors, and improves both longitudinal and lateral stability of the vehicle. This study provides an effective solution for enhancing the active safety control of in-wheel motor driven electric vehicles operating in icy and snowy environments.

## 1. Introduction

Affected by climate change, driving safety under extreme weather conditions has become increasingly prominent. Particularly in severe winter regions, roads are often covered with snow and ice, which form complex low-friction surfaces after compaction. These surfaces readily induce wheel spin, vehicle sideslip, fishtailing, and steering instability. This critical situation poses new challenges to vehicle ASR performance [1–3]. As a new generation of intelligent transportation systems, the in-wheel motor driven electric vehicle provides a novel technical approach to address ASR challenges through its unique driving architecture and rapid response characteristics.

Compared with conventional centralized-drive vehicles, the in-wheel motor driven electric vehicle achieves independent and precise torque control for each driving wheel, providing superior performance in traction, braking, acceleration slip regulation (ASR), and stability control [4–7]. However, due to the extremely low and distinctive friction coefficient of compacted snow surfaces [8–10], tires may fail to recover sufficient traction during acceleration or startup, resulting in excessive wheel slip and spin. This further deteriorates road adhesion conditions, severely compromising vehicle mobility and safety. Therefore, the ASR control strategy designed for the in-wheel motor driven electric vehicle operating on compacted snow surfaces must incorporate proactive slip prevention to avoid excessive wheel spin, fully account for the impacts of tire-snow friction characteristics, while effectively maintaining vehicle driving stability.

At present, the ASR control strategies for in-wheel motor driven electric vehicle can be primarily categorized into two types based on their core principles. The first category focuses on tracking the optimal slip ratio. For instance, Zhang Z et al. [11] employed a fading-memory unscented Kalman filter to estimate the road adhesion coefficient and derived the optimal slip ratio based on the relationship curve between the road adhesion coefficient and the slip ratio, applying it to anti-slip control to improve vehicle passability. Abbas Soltani et al. [12] proposed an integrated control strategy based on optimal wheel slip ratio estimation. This scheme employs an Unscented Kalman Filter (UKF) to estimate key state variables in real time for calculating the road friction coefficient, and introduces an Adaptive Neuro-Fuzzy Inference System (ANFIS) to predict the optimal wheel slip ratio under different road conditions. By coordinating the Active Front Steering (AFS) and Active Braking (AB) systems, the approach comprehensively enhances the longitudinal, lateral, and yaw stability of vehicles during emergency braking scenarios. Zhang HZ et al. [13] estimated the road adhesion coefficient using the recursive least squares method, determined the optimal slip ratio via the Burckhardt tire model, and achieved rapid tracking through a sliding mode controller. Zhang LP et al. [14] combined the relationship curve between tire longitudinal force and wheel slip ratio with a variable β sliding mode extremum seeking algorithm to calculate the optimal slip ratio in real time, thereby solving for the desired driving torque. These control methods, which aim to track the optimal slip ratio to maximize traction force, have achieved effective acceleration slip regulation (ASR) performance on conventional road surfaces. However, their core reliance on accurate identification of road adhesion coefficient and real-time calculation of optimal slip ratio inevitably increases the complexity of the control strategy and introduces inherent delays in identification and computation. The second category focuses more on maintaining vehicle driving stability. Some studies in this category primarily concentrated on longitudinal stability control. For example, Bao YT [15] and Sun XM [16] considered longitudinal stability when designing their acceleration slip regulation (ASR) strategies, emphasizing vehicle longitudinal anti-slip performance. However, they neglected lateral stability control and did not account for steering conditions in experimental validation, making them inadequate for complex and varying real-world driving conditions. Some researchers have also attempted to achieve coordinated control of both longitudinal and lateral stability. For example, Wu JY [17] et al. adopted a coordinated control strategy integrating acceleration slip regulation (ASR) with direct yaw moment control (DYC), utilizing priority evaluation and multi-objective coordination to simultaneously enhance both longitudinal and lateral vehicle stability. However, this priority-based control method may cause torque abruptions during logic switching between control modules, making it difficult to simultaneously maintain stability in both directions.

Liang JH [18] et al. applied Pareto optimal theory to identify the optimal operating ranges for active front steering (AFS) and direct yaw moment control (DYC), thus achieving integrated longitudinal and lateral vehicle stability control. Dang M [19] et al. developed a coordinated control strategy between AFS and differential drive assisted steering (DDAS) systems to mitigate undesired yaw moment effects, with AFS/DDAS working regions demarcated based on sideslip angle deviation. Liang JH et al. [20] proposed a cooperative control framework that integrates torque vectoring (TV) and an active front steering (AFS) system. This scheme treats TV and AFS as two interactive intelligent agents. Using distributed model predictive control (DMPC) and game theory, the agents pursue their respective control objectives while achieving cooperative optimization. Terminal constraints are introduced to ensure the asymptotic stability of the system. Zheng, ZC [21] et al. established an evolutionary game model to coordinate AFS and DYC control weight distribution across various vehicle operating states, leading to enhanced driving stability. Liang JH et al. [22] employed T-S fuzzy rules to handle the nonlinear characteristics of tires and constructed a vehicle stability region represented by the phase plane of the tire sideslip angle. The scheme dynamically adjusts the priority between handling performance and stability control based on the stability margin. Thereby, the multi-objective control problem is transformed into a robust H∞ performance optimization problem with dynamic weights, achieving an effective balance between vehicle handling and stability. Li Q [23] et al. implemented phase plane analysis to delineate the working regions of DDAS and vehicle stability control (VSC) systems, with their coordinated operation improving both vehicle maneuverability and stability. These studies improved vehicle stability under complex driving conditions by partitioning operational domains or coordinating control weights to schedule different control methods. However, they generally neglected the influence of wheel slip ratio on driving stability. Control strategies that rely solely on wheel steering or torque redistribution lack effective constraints on slip ratio, which can easily cause excessive slip of individual wheels and consequently impair vehicle stability.

In summary, although acceleration slip regulation (ASR) for in-wheel motor driven electric vehicles has been extensively studied, existing methods still exhibit significant limitations when dealing with compacted snow surfaces. Firstly, control methods aimed at pursuing the optimal slip ratio struggle to effectively suppress excessive wheel slip and fail to efficiently utilize limited road adhesion. Moreover, their reliance on parameter identification tends to cause computational delays. Secondly, there is inadequate coordination between longitudinal anti-slip control and lateral stability control, with control logic transitions prone to inducing torque abruptions. Finally, most current coordinated longitudinal-lateral control strategies neglect slip ratio constraints, relying solely on steering or torque redistribution, which may lead to excessive wheel slip and consequently compromise stability.

Therefore, to efficiently utilize the limited tire-compacted snow friction and achieve coordinated control of both longitudinal slip prevention and lateral stability, while simplifying the control architecture and reducing time delays, this paper proposes a dynamically slip-ratio-constrained longitudinal-lateral coordinated optimal acceleration slip regulation (ASR) control strategy. The main contributions of this study are as follows:

(1) A “dynamic slip ratio constraint” control method is proposed, which proactively confines the slip ratio within a lower, safe range. This ensures a sufficient reserve of tire lateral force and significantly enhances the vehicle’s anti-skid capability.
(2) Achieving deep coordination between acceleration slip regulation and active steering, rather than a simple superposition of control modules. By establishing a feedforward-feedback composite control architecture, the two systems work together coordinatively, avoiding disturbances caused by control mode switching and realizing a seamless integration of longitudinal and lateral stability.
(3) A multi-objective real-time optimization function based on dynamic slip ratio constraints was constructed. This strategy can dynamically adjust the slip status of all four wheels according to complex operating conditions (e.g., low adhesion, steep slopes, accelerated turning), comprehensively balancing dynamics, anti-slip performance, and stability. Thereby, while suppressing wheel slip, it provides a more stable controlled object for the subsequent AFS feedback control. Co-simulation results using Carsim and Matlab/Simulink demonstrate that this strategy effectively suppresses wheel slip and reduces trajectory deviation, providing a practical solution for the safety control of in-wheel motor electric vehicles on icy and snowy roads.

## 2. Vehicle dynamic model

Taking in-wheel motor driven electric vehicle as the research object, a seven-degree-of-freedom (7-DOF) vehicle dynamics model, Magic Formula tire model, and two-degree-of-freedom (2-DOF) vehicle dynamics reference model were established to provide the foundation for subsequent simulation experiments [24–26].

### 2.1. Vehicle dynamics equation

**Fig 1:**
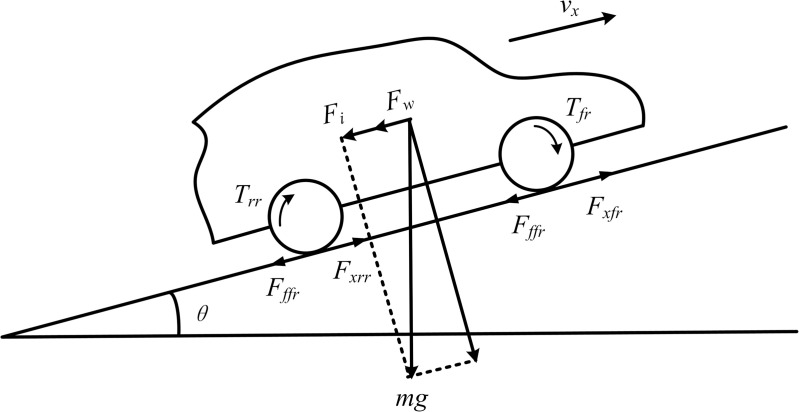
Force analysis of vehicles on slopes.


The force analysis of the vehicle on a longitudinal slope is shown in Fig 1, and the vehicle’s force equilibrium equation is expressed by Equation (1):

$$\left\{ \begin{array}{c} F_{x}=F_{f}+F_{w}+F_{i}+F_{j}\\ F_{x}=\frac{T_{fl}+T_{fr}+T_{rl}+T_{rr}}{r}\\ F_{f}=fmg\cos\theta \\ F_{w}=\frac{C_{d}A{v_{x}}^{2}}{21.15}\\ F_{i}=mg\sin\theta \end{array}\right.$$ (1)

In the equation, *F*_*x*_ represents the longitudinal driving force; *F*_*f*_ represents the rolling resistance; *F*_*w*_ represents the air resistance; *F*_*i*_ represents the gradient resistance; *F*_*j*_ represents the acceleration resistance; *T*_*ij*_ represents the drive torque of each wheel (where *ij* = *fl*, *fr*, *rl*, *rr*; representing the front-left wheel, front-right wheel, rear-left wheel, and rear-right wheel, respectively); *r* represents the tire rolling radius; *f* represents the rolling resistance coefficient; *θ* represents the road slope; *C*_*d*_ represents the drag coefficient; *A* is the frontal area; *v*_*x*_ is the longitudinal vehicle speed.

Consequently, the necessary driving torque to achieve the target longitudinal acceleration *a*_*x*_ is obtained as:


$$T_{M}=(fmg\cos\theta +\frac{C_{d}A{v_{x}}^{2}}{21.15}+mg\sin\theta +ma_{x})r$$ (2)

### 2.2. 7-DOF vehicle dynamics model

**Fig 2:**
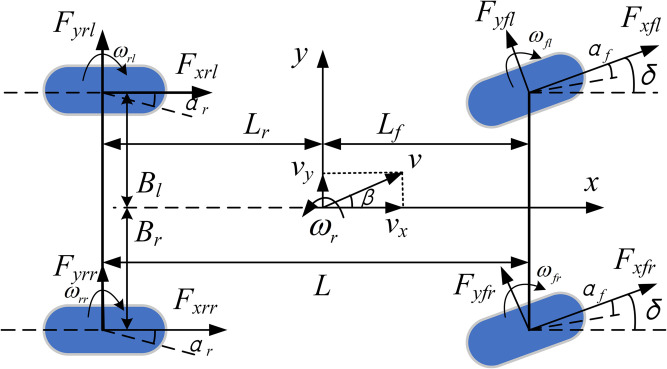
7-DOF vehicle dynamics model.

To acquire the vehicle’s state parameters during operation, ignoring the effects of the vehicle suspension system, as well as the vehicle’s roll and pitch motions; assuming the vehicle body is a rigid body; assuming all in-wheel motors and tires have identical characteristics; and considering slope driving conditions, a 7-DOF vehicle dynamics reference model is established, as shown in Fig 2. This model includes three degrees of freedom (longitudinal, lateral, and yaw motions) and four rotational degrees of freedom for the wheels.

Thus, the 7-DOF vehicle dynamics equations can be derived as follows:


$$ma_{x}=m({\overset{˙}{v}}_{x}-\omega_{r}v_{y})=(F_{xfl}+F_{xfr})cos\delta -F_{f}-(F_{yfl}+F_{yfr})sin\delta +F_{xrl}+F_{xrr}-F_{i}-F_{w}$$ (3)

$$ma_{y}=m({\overset{˙}{v}}_{y}+\omega_{r}v_{x})=(F_{xfl}+F_{xfr})sin\delta +(F_{yfl}+F_{yfr})cos\delta \;+F_{yrl}+F_{yrr}$$ (4)

$$\begin{array}{c} I_{z}{\dot{\omega }}_{r}=L_{f}[(F_{yfl}+F_{yfr})\cos\delta +(F_{xfl}+F_{xfr})\sin\delta ]-L_{r}(F_{yrl}+F_{yrr})\\ \;\;+B_{l}(F_{yfl}\sin\delta -F_{xfl}\cos\delta -F_{xrl})+B_{r}(F_{xfr}\cos\delta -F_{yfr}\sin\delta +F_{xrr}) \end{array}$$ (5)

In the equations, *v*_*x*_ and *v*_*y*_ represent the longitudinal and lateral vehicle speeds, respectively; *a*_*x*_ and *a*_*y*_ represent the longitudinal and lateral accelerations, respectively; *F*_*xij*_ denotes the longitudinal force of each wheel; *F*_*yi*j_ denotes the lateral force of each wheel; *δ* is the front wheel steering angle; *I*_*z*_ is the vehicle’s moment of inertia about the Z-axis; *ω*_*r*_ is the yaw rate; *L*_*f*_ and *L*_*r*_ are the distances from the front and rear axles to the center of mass, respectively; *B*_*l*_ and *B*_*r*_ are the distances from the left and right wheels to the center of mass, respectively; *β* is the sideslip angle of the center of mass; *α*_*f*_ and *α*_*r*_ are the slip angles of the front and rear wheels, respectively.

**Fig 3:**
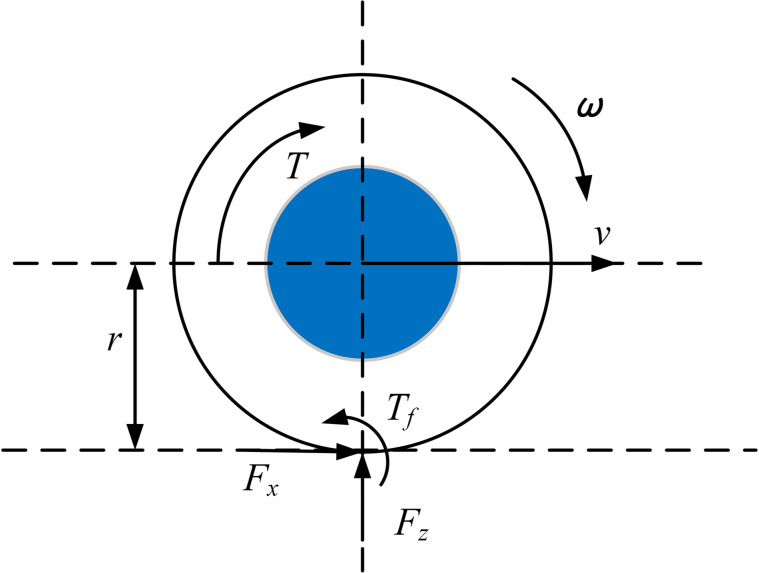
Wheel rotational dynamics model.

The rotational dynamics model of the wheel is shown in Fig 3:

Thus, the wheel rotational dynamics equation can be obtained:


$$I_{w}\frac{d\omega_{ij}}{dt}=T_{ij}-F_{xij}r-T_{fij}$$ (6)

$$T_{fij}=F_{zij}\cdot f\cdot r$$ (7)

In the equations, *I*_*w*_ is the wheel’s moment of inertia; *ω*_*ij*_ represents the angular velocity of each wheel; *T*_*ij*_ denotes the driving torque of each wheel; *F*_*fij*_ is the rolling resistance of each wheel; *T*_*fij*_ is the rolling resistance moment of each wheel; and *F*_*zij*_ represents the vertical force of each wheel.

Ignoring the influence of the suspension, the vertical load of each wheel is calculated as follows:


$$\begin{array}{c} \left[\begin{array}{c} {}*20cF_{zfl}\\ F_{zfr}\\ F_{zrl}\\ F_{zrr} \end{array}\right]=\left[\begin{array}{c} {}*20cL_{r}B_{r}\\ L_{r}B_{l}\\ L_{f}B_{r}\\ L_{f}B_{l} \end{array}\right]\cdot \frac{mg\cos\theta }{LB}+\left[\begin{array}{c} {}*20c-L_{r}B_{r}\\ -L_{r}B_{l}\\ L_{f}B_{r}\\ L_{f}B_{l} \end{array}\right]\cdot \frac{mgh_{g}\sin\theta }{LB}\\ +\left[\begin{array}{c} {}*20c-1\\ -1\\ 1\\ 1 \end{array}\right]\cdot \frac{ma_{x}h_{g}}{2L}+\left[\begin{array}{c} {}*20c-L_{r}\\ L_{r}\\ -L_{f}\\ L_{f} \end{array}\right]\cdot \frac{ma_{y}h_{g}}{LB} \end{array}$$ (8)

The slip ratio λ is a key variable that characterizes the degree of wheel slip, and its calculation formula is:


$$\lambda =\frac{\omega \cdot r-v_{x}}{\omega \cdot r}\cdot 100\%$$ (9)

The relationship between the slip angles of the front and rear wheels and the steering angle of the front wheels is:


$$\left\{ \begin{array}{c} \alpha_{f}=\delta -\beta -\frac{L_{f}\omega_{r}}{v_{x}}\\ \alpha_{r}=-\beta +\frac{L_{r}\omega_{r}}{v_{x}} \end{array}\right.$$ (10)

### 2.3. Tire model

As the only contact point between the vehicle and the road, the mechanical characteristics of tires directly affect the handling stability and safety of the vehicle. To better reflect the real operating state of the vehicle, this paper adopts the Magic Formula tire model to calculate the longitudinal and lateral forces acting on the tires. The expression is as follows:


$$\left\{ \begin{array}{c} y(x)=D\sin\{C\arctan[Bx-E(Bx-\arctan(Bx))\}\\ Y(x)=y(x)+S_{v}\\ x=X+S_{h} \end{array}\right.$$ (11)

In the equation, *Y(x)* represents the longitudinal force, lateral force, or aligning torque; *X* represents the slip ratio or slip angle; *B* represents the stiffness factor; *C* represents the curve shape factor; *D* represents the peak factor; *E* represents the curvature factor; *S*_*v*_ represents the vertical shift of the curve; and *S*_*h*_ represents the horizontal shift of the curve.

### 2.4 Motor model

The drive motor, serving as the core actuator in electric vehicles, significantly influences overall vehicle control performance through the dynamic characteristics of its output torque. A permanent magnet synchronous motor is selected as the drive motor, and the relationship between its output torque *T*_*out*_ and the target torque *T*_*in*_ can be represented by the following transfer function:


$$G(s)=\frac{T_{out}}{T_{in}}=\frac{\text{1}}{1+2\zeta s+2\zeta^{2}s^{2}}$$ (12)

Where *T*_*in*_ is the target torque calculated by the optimization-based allocation controller, *T*_*out*_ is the actual torque output by the motor, and ζ is the damping coefficient determined by the motor parameters.

### 2.5. 2-DOF vehicle dynamics reference model

**Fig 4:**
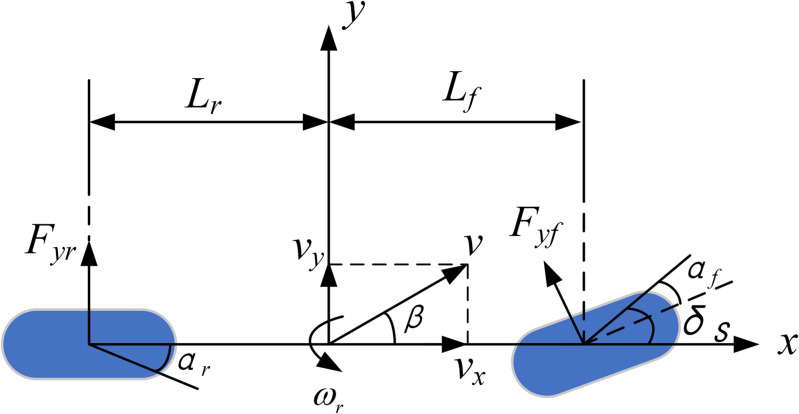
Linear 2-DOF vehicle dynamics reference model.

The linear 2-DOF vehicle model (as shown in Fig 4) has a simple structure and involves fewer vehicle characteristic parameters, yet it can effectively reflect the relationship between the wheel steering angle and the sideslip angle as well as the yaw rate. Therefore, the linear 2-DOF model is adopted as the reference model for designing the controller to calculate the ideal yaw rate and the ideal sideslip angle during vehicle operation.

In Fig 4, *F*_*yf*_ and *F*_*yr*_ represent the lateral forces of the front and rear axles, respectively, and *δ*_*s*_ denotes the steering angle of the front wheels under the action of the steering wheel. Thus, the differential equations of the 2-DOF model can be derived as follows:


$$\left\{ \begin{array}{c} {\dot{\omega }}_{r}=(\frac{L_{f}k_{f}-L_{r}k_{r}}{I_{z}})\beta +(\frac{{L_{f}}^{2}k_{f}+{L_{r}}^{2}k_{r}}{I_{z}v_{x}})\omega_{r}-\frac{L_{f}k_{f}}{I_{z}}\delta_{s}\\ \dot{\beta }=(\frac{k_{f}+k_{r}}{mv_{x}})\beta +(\frac{L_{f}k_{f}-L_{r}k_{r}}{m{v_{x}}^{2}}-1)\omega_{r}-\frac{k_{f}}{mv_{x}}\delta_{s} \end{array}\right.$$ (13)

In the equation, *k*_*f*_ and *k*_*r*_ represent the cornering stiffness of the front and rear axles, respectively. To facilitate subsequent derivations, Equation (13) is rewritten in state-space form as follows:


$$\left[\begin{array}{c} {\dot{\omega }}_{r}\\ \dot{\beta } \end{array}\right]=\left[\begin{array}{cc} {}*20ca_{11} & a_{12}\\ a_{21} & a_{22} \end{array}\right]\left[\begin{array}{c} {}*20c\beta \\ \omega_{r} \end{array}\right]+\left[\begin{array}{c} {}*20cb_{1}\\ b_{2} \end{array}\right]\delta_{s}$$ (14)

In the equation: a_11_=(*L*_*f*_*k*_*f*_-*L*_*r*_*k*_*r*_)/*I*_*z*_, a_12_=(*L*_*f*_^2^*k*_*f*_ + *L*_*r*_^2^*k*_*r*_)/*I*_*z*_*v*_*x*_, a_21_=(*k*_*f*_ + *k*_*r*_)/*mv*_*x*_, a_22_=(*L*_*f*_*k*_*f*_-*L*_*r*_*k*_*r*_)/*mv*_*x*_^2^−1, b_1_=-*L*_*f*_*k*_*f*_/*I*_*z*_, b_2_=-*k*_*f*_/*mv*_*x*_.

The ideal yaw rate and sideslip angle of the vehicle can be derived from the linear 2-DOF vehicle model. However, during actual driving conditions, these ideal values are constrained by the road adhesion coefficient. Based on empirical formulas, the ideal yaw rate can be expressed as:


$$\omega_{d}=\min\{\left|\frac{v}{(1+K{v_{x}}^{2})L}\delta_{s}\right|,\left|0.85\frac{\mu g}{v_{x}}\right|\}sgn(\delta_{s})$$ (15)

In the equation, *L* represents the wheelbase; *ω*_*d*_ represents the desired yaw rate of the vehicle; and *K* represents the stability factor, whose expression is as follows:


$$K=\frac{m}{L^{2}}(\frac{L_{f}}{k_{r}}-\frac{L_{r}}{k_{f}})$$ (16)

From the perspective of vehicle safety and stability, an excessive sideslip angle must be avoided to prevent vehicle skidding. Therefore, the ideal sideslip angle is selected as zero. That is:


$$\beta_{d}=0$$ (17)

## 3. Coordinated control of ASR and AFS

**Fig 5:**
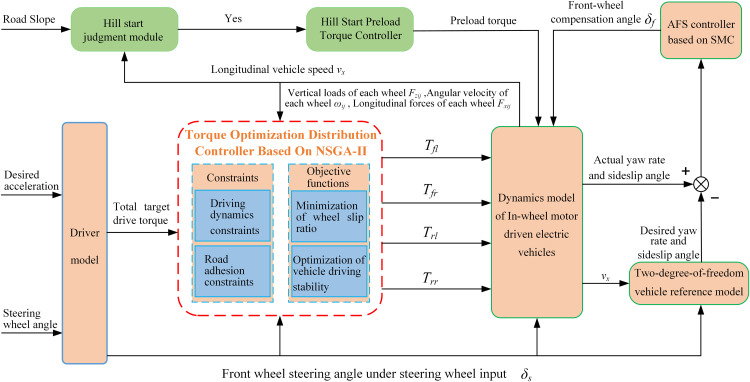
Overall structure of the coordinated control strategy.

On the basis of obtaining the total target driving torque, a coordinated control strategy for drive anti-slip and active front steering (AFS) based on multi-objective optimization was designed [27–29]. The control framework is shown in Fig 5. The strategy includes a drive torque optimal distribution module, which utilizes dynamic slip ratio constraints and the NSGA-II algorithm. While meeting the total driving demand, it actively limits the slip ratio of each wheel within a safe range, minimizes slip loss, and preliminarily coordinates wheel speeds to suppress undesired yaw moments. AFS Compensation Module: Based on sliding mode control (SMC), it takes the desired yaw rate and sideslip angle as tracking targets to dynamically generate front-wheel compensation angles. This precisely counteracts undesired yaw moments caused by road unevenness or torque distribution itself, thereby enhancing directional stability. The two modules work synergistically to form a closed-loop system, achieving efficient coordination between drive anti-slip and directional stability. Additionally, a pre-torque control strategy is specifically developed for hill-start conditions to ensure smooth launching.

### 3.1 NSGA-II-based ASR controller

**Fig 6:**
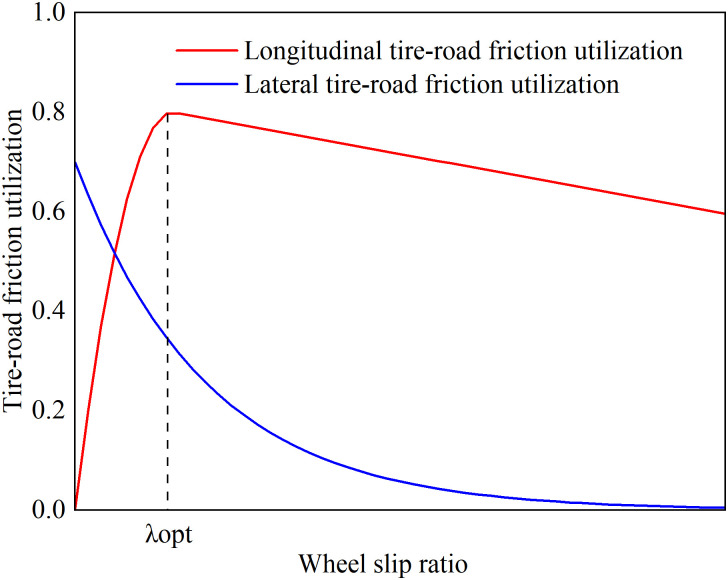
Tire-road adhesion characteristic curve.

The tire-road adhesion characteristic curve shown in Fig 6 indicates that tracking the optimal slip ratio aims to maximize the longitudinal utilized adhesion coefficient under current road conditions, thus achieving maximum longitudinal traction. However, this approach significantly reduces the tire’s lateral force reserve, making the vehicle prone to skidding or spin instability during steering maneuvers. Additionally, excessive slip exacerbates the frictional thermal effects between the tire and snow, compromising the limited contact stability of the snow surface and further reducing the road adhesion coefficient. Therefore, when driving on compacted snow, it is necessary to dynamically adjust the slip ratio constraints to ensure vehicle dynamics while limiting the slip ratio within a smaller safety range, thereby improving lateral stability.

Therefore, an ASR controller based on NSGA-II [30–31] was designed. Its control inputs include: the total target driving torque *T*_*M*_; the vertical forces on each wheel (*F*_*zfl*_, *F*_*zfr*_, *F*_*zrl*_, *F*_*zrr*_); the road adhesion coefficients at the contact patch of each wheel (*μ*_*fl*_, *μ*_*fr*_, *μ*_*rl*_, *μ*_*rr*_); the longitudinal vehicle velocity *v*_*x*_; the angular velocities of each wheel (*ω*_*fl*_, *ω*_*fr*_, *ω*_*rl*_, *ω*_*rr*_); the longitudinal forces on each wheel (*F*_*xfl*,_ *F*_*xfr*_, *F*_*xrl*_, *F*_*xrr*_); and the front wheel steering angle under steering wheel input *δ*_*f*_. With the objectives of minimizing the wheel slip ratio and maintaining vehicle stability, the driving torque is optimally distributed to all four wheels to achieve acceleration slip regulation for the entire vehicle, thereby ensuring its driving mobility and stability.

#### 3.1.1. Constraint conditions.

The sum of the distributed drive torques must always equal the total target drive torque *T*_*M*_. That is:


$$T_{fl}+T_{fr}+T_{rl}+T_{rr}=T_{M}$$ (18)

The distributed drive torque for each wheel must satisfy the road adhesion conditions. That is:


$$T_{ij}\leq (\mu_{ij}+f)\cdot F_{zij}\cdot r$$ (19)

In the equation, *μ*_*ij*_ represents the road adhesion coefficient at the contact point of each wheel.

#### 3.1.2. Objective functions.

When driving on compacted snow surfaces, excessive slip of any single wheel can lead to excessive friction between the tire and the compacted snow surface, further reducing the road adhesion coefficient and the vehicle’s driving dynamics, affecting the vehicle’s mobility, and introducing potential safety risks. Therefore, it is necessary to minimize the slip ratio of each wheel while meeting the vehicle’s dynamic performance requirements. The objective function is as follows:


$$\left\{ \begin{array}{c} \min(f_{1})=\lambda_{fl}+\lambda_{fr}+\lambda_{rl}+\lambda_{rr}\\ \min(f_{2})=(\lambda_{fl}-\bar{\lambda }{)}^{2}+(\lambda_{fr}-\bar{\lambda }{)}^{2}+(\lambda_{rl}-\bar{\lambda }{)}^{2}+(\lambda_{rr}-\bar{\lambda }{)}^{2} \end{array}\right.$$ (20)

In the equation, *λ*_*ij*_$\bar{\lambda }$ represents the average slip ratio of the four wheels.

During straight-line driving, in order to reduce phenomena such as vehicle sideslip and fishtailing, and to maintain driving stability, it is necessary to minimize the speed difference among the four wheels while meeting the vehicle’s dynamic performance requirements. The objective function is as follows:


$$\min(f_{3})=(v_{fl}-\bar{v}{)}^{2}+(v_{fr}-\bar{v}{)}^{2}+(v_{rl}-\bar{v}{)}^{2}+(v_{rr}-\bar{v}{)}^{2}$$ (21)

In the equation, $\bar{v}$ is the average speed of the four wheels.

**Fig 7:**
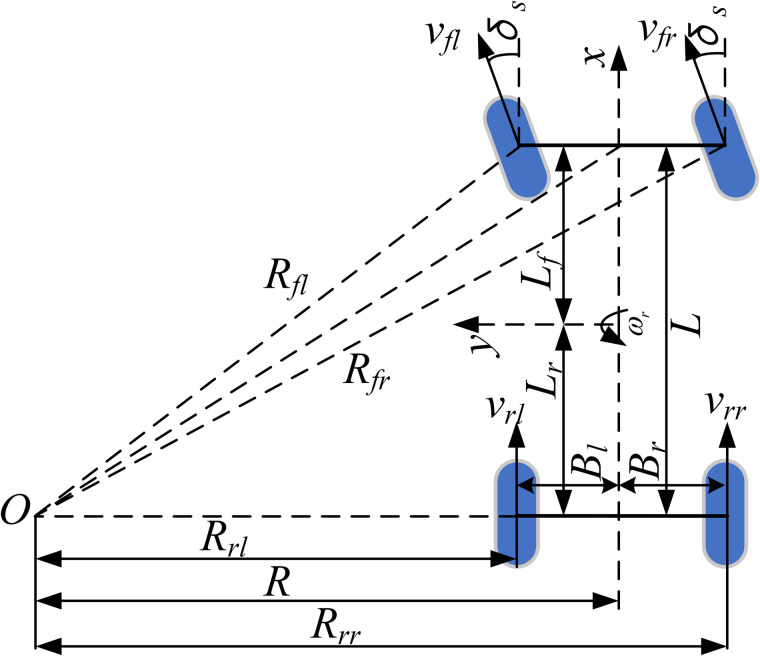
Ackermann steering model.

During cornering, to meet the speed difference requirements between the inner and outer wheels and to prevent wheel slip on the inner side, each wheel’s speed should be controlled to approach its corresponding optimal speed. According to the Ackermann steering geometry (as illustrated in Fig 7), all wheels should ideally rotate around the same instantaneous center of rotation (ICR) under perfect conditions.

In the figure, *v*_*ij*_ represents the actual speed of each wheel, and *R*_*ij*_ denotes the instantaneous turning radius of each wheel.

Ignoring the wheel slip angles, the vehicle’s turning radius can be expressed as:


$$R=\frac{L}{\tan\left|\delta_{s}\right|}$$ (22)

Therefore, when the vehicle turns left (*δ*_*s*_ > 0), the instantaneous turning radius of each wheel is given by:


$$\left\{ \begin{array}{c} R_{fl}=\sqrt{{\left(R-B_{l}\right)}^{2}+L^{2}}\\ R_{fr}=\sqrt{{\left(R+B_{r}\right)}^{2}+L^{2}}\\ R_{rl}=R-B_{l}\\ R_{rr}=R+B_{r} \end{array}\right.$$ (23)

When the vehicle turns right (*δ*_*s*_ < 0), the instantaneous turning radius of each wheel is given by:


$$\left\{ \begin{array}{c} R_{fl}=\sqrt{{\left(R+B_{l}\right)}^{2}+L^{2}}\\ R_{fr}=\sqrt{{\left(R-B_{r}\right)}^{2}+L^{2}}\\ R_{rl}=R+B_{l}\\ R_{rr}=R-B_{r} \end{array}\right.$$ (24)

According to rigid-body kinematics, when the vehicle turns about the instantaneous center of rotation (ICR), the linear velocity of each wheel is proportional to its turning radius. Therefore, the ideal wheel speeds are given by:


$$\left\{ \begin{array}{c} {v_{fl}}_{\_\exp}=\frac{v_{x}\cdot \sqrt{{\left(R-B_{l}\right)}^{2}+L^{2}}}{R}\\ {v_{fr}}_{\_\exp}=\frac{v_{x}\cdot \sqrt{{\left(R+B_{r}\right)}^{2}+L^{2}}}{R}\\ v_{rl\_\exp}=\frac{v_{x}\cdot (R-B_{l})}{R}\\ {v_{rr}}_{\_\exp}=\frac{v_{x}\cdot (R+B_{r})}{R} \end{array}\right.$$ (25)

To summarize, the design objective function during cornering is formulated as:


$$\min(f_{3})=(v_{fl}-{v_{fl}}_{\_\exp}{)}^{2}+(v_{fr}-{v_{fr}}_{\_\exp}{)}^{2}+(v_{rl}-{v_{fl}}_{\_\exp}{)}^{2}+(v_{rr}-{v_{rr}}_{\_\exp}{)}^{2}$$ (26)

To improve adhesion utilization efficiency, prevent wheel spin due to insufficient load on individual wheels, and avoid the multi-objective genetic optimization algorithm converging to local optima, the drive torque should be distributed proportionally to the wheel loads. This ensures each wheel’s driving force matches its weight bearing capacity. The designed optimization objective function is as follows:


$$\min(f_{4})=(\frac{T_{fl}}{T_{M}}-\frac{F_{zfl}}{F_{zh}}{)}^{2}+(\frac{T_{fr}}{T_{M}}-\frac{F_{zfr}}{F_{zh}}{)}^{2}+(\frac{T_{rl}}{T_{M}}-\frac{F_{zrl}}{F_{zh}}{)}^{2}+(\frac{T_{rr}}{T_{M}}-\frac{F_{zrr}}{F_{zh}}{)}^{2}$$ (27)

In the equation, *F*_*zh*_ represents the sum of the vertical loads of the four wheels.

#### 3.1.3. Drive torque optimization distribution.

Based on the defined constraints and objective function, the NSGA-II is employed to optimize the total drive torque allocation [32–34]. The implementation workflow consists of the following key steps:

Step 1: Hybrid Initialization Population. To improve initial population quality for generating superior solutions while avoiding local optima and maintaining population diversity, a hybrid initialization strategy is implemented as follows: 60% randomly generated individuals to ensure global search capability, 10% historically optimal solutions, and 30% solutions based on load distribution ratios with ±5% random perturbations, thereby guiding the creation of a high-quality initial population.

Step 2: Fitness Calculation. Calculate the objective function value for each individual by combining Equations (6) and (7). To avoid misjudging vehicle steering status due to small wheel angles (which would cause frequent switching of the objective function min(*f*₃)), steering conditions are only considered when the angle exceeds 0.002 rad. To eliminate the influence of different units of measurement among the four objective functions, a normalization formula is applied to dynamically normalize each objective function, ensuring that each function is mapped to the range [0, 1]. The sum of the normalized values of the four objective functions is taken as the fitness value.


$$N^{*}=\frac{N-N_{\min}}{N_{\max}-N_{\min}}$$ (28)

In the formula, N* represents the normalized data, while *N*_*max*_ and *N*_*min*_ denote the maximum and minimum values of the objective function in the current population, respectively.

Step 3: Fast Non-dominated Sorting Each individual in the population is stratified based on Pareto dominance relations. Non-dominated individuals are assigned to the first front. After removing this set, the process is repeated for the remaining individuals until the entire population is stratified.

Step 4: Crowding Distance Calculation For individuals within each front, sort them in ascending order according to each objective value. Assign an infinite crowding distance to the boundary individuals (the first and last individuals in the sorted list). For the remaining intermediate individuals, the crowding distance is computed as the sum of the absolute normalized distances between their adjacent neighbors along each objective.

Step 5: Elite Parent Selection and Offspring Generation. Select elite parents from the current population, then generate a new offspring population through crossover and mutation operations. Notably, the mutation probability adaptively decreases as the iteration count increases.

Step 6: Population Merging and Iteration. Combine the parent and offspring populations, then repeat Steps 2–5 for the next iteration.

Step 7: Termination Condition Check. If the maximum iteration count is reached, the algorithm terminates and outputs the optimal solutions satisfying all constraints. Otherwise, Steps 2–5 are repeated until the termination criterion is met.

**Fig 8:**
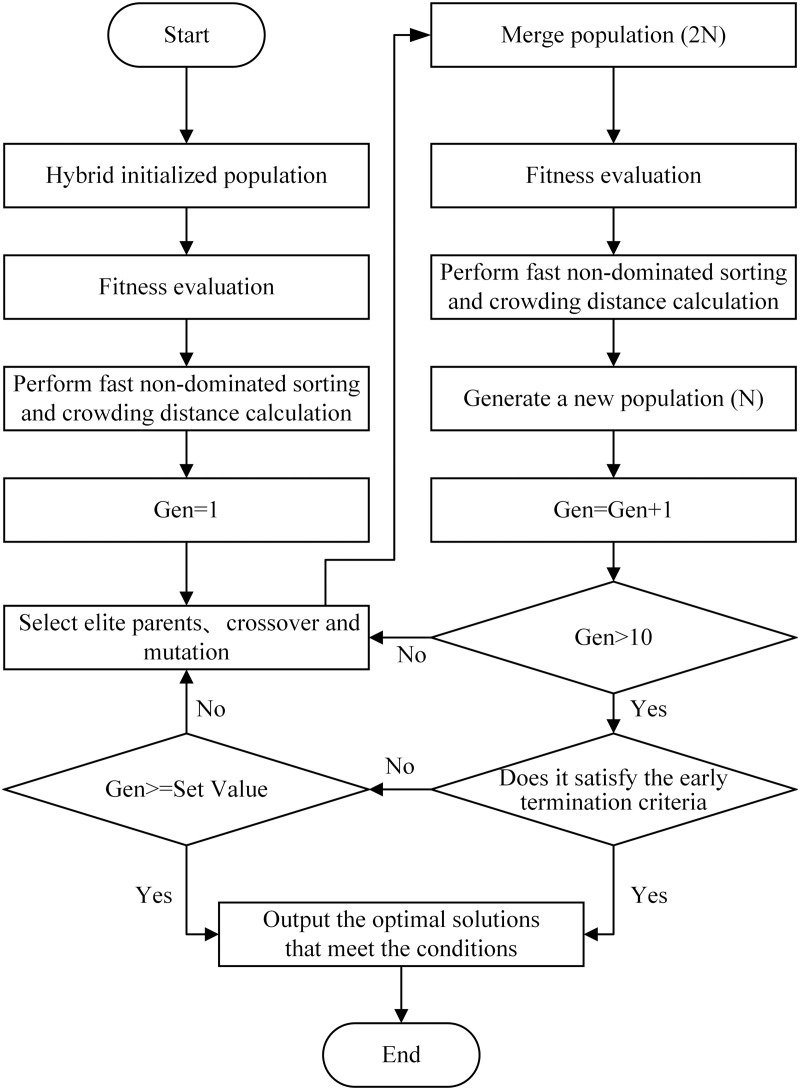
Flowchart of the NSGA-II.

Additionally, to avoid redundant computations, an early termination mechanism is established. It records the best-fit individuals from the most recent 10 generations and defines the fitness change as the absolute difference between the current best fitness value and the historical best fitness value. If the standard deviation of the best fitness changes remains below 0.01 for five consecutive generations, the termination condition is triggered, and the current best individual is output. A detailed flowchart is illustrated in Fig 8.

Using the NSGA-II, the total target drive torque can be dynamically optimized and distributed, thereby obtaining the target torque *T*_*ij*_ for all four wheels under the current vehicle driving conditions.

#### 3.1.4. Preload torque for hill start.

Due to the inherent hysteresis in the actual torque response of in-wheel motors, vehicles are prone to rolling backward when starting on a slope. To effectively address this issue, an adaptive preload torque control strategy for hill starts is designed. This strategy ensures smooth hill starts by controlling the braking torque of the vehicle’s brakes. The controller activates when the road slope *θ* > 0 and the vehicle speed *v* = 0. The required preload torque *Ty* for hill starts is calculated based on the road slope *θ* and the longitudinal tire forces of all four wheels. The preload torque is deactivated when the vehicle’s longitudinal acceleration *a*_*x*_ exceeds 0. Thus, the designed preload torque is expressed as:


$$T_{y}=\left\{ \begin{array}{c} {}[F_{f}+F_{i}-(F_{xfl}+F_{xfr}+F_{xrl}+F_{xrr})]r\\ F_{f}+F_{i}>F_{xfl}+F_{xfr}+F_{xrl}+F_{xrr}\\ 0\\ F_{f}+F_{i}\leq F_{xfl}+F_{xfr}+F_{xrl}+F_{xrr} \end{array}\right.$$ (29)

According to Equation (27), the applied preload torque is used to balance the driving resistance on a slope. When the vehicle’s longitudinal acceleration *a*_*x*_ exceeds 0, it indicates that the driving force has overcome the resistance, and the preload torque is subsequently released to ensure smooth vehicle starting.

Meanwhile, to balance the adhesion utilization of each wheel and prevent slip on lightly loaded wheels, the total preload torque is distributed to individual wheels based on their vertical loads. Combining Equation (8), the preload torque to be applied to each wheel is given by:


$$\left\{ \begin{array}{c} T_{yfl}=\frac{F_{zfl}}{F_{zh}}\cdot T_{y}\\ T_{yfr}=\frac{F_{zfr}}{F_{zh}}\cdot T_{y}\\ T_{yrl}=\frac{F_{zrl}}{F_{zh}}\cdot T_{y}\\ T_{yrr}=\frac{F_{zrr}}{F_{zh}}\cdot T_{y} \end{array}\right.$$ (30)

### 3.2. SMC-based AFS compensation controller

To further enhance the vehicle’s directional stability, an active front steering (AFS) compensation controller was designed using a sliding mode control (SMC) algorithm. The controller takes the desired yaw rate *ω*_*d*_, the desired sideslip angle *β*_*d*_, the actual yaw rate *ω*_*r*_, the actual sideslip angle *β*, and the longitudinal velocity *v*_*x*_ as its control inputs. It outputs a front-wheel compensation angle *δ*_*f*_, which works in coordination with the acceleration slip regulation (ASR) system to maintain vehicle stability [35–36].

#### 3.2.1. Sliding mode control strategy design.

According to the linear two-degree-of-freedom vehicle reference model, the desired yaw rate and sideslip angle during vehicle motion can be obtained. The sliding mode control error is defined as the difference between the desired state parameters and the actual state parameters, that is:


$$\left\{ \begin{array}{c} e_{1}=\omega_{r}-\omega_{d}\\ e_{2}=\beta -\beta_{d} \end{array}\right.$$ (31)

An intermediate variable *Z* is introduced for the design of the sliding surface, enabling integrated control of the yaw rate and sideslip angle.


$$Z=e_{1}+\xi e_{2},\xi =0.8$$ (32)

The sliding surface *S* and its derivative are defined, where *c* > 0 satisfies the Hurwitz condition:


$$\left\{ \begin{array}{c} S=Z+\int_{0}^{t}Z(t)dt\\ \dot{S}=\dot{Z}+cZ \end{array}\right.$$ (33)

By substituting Equations (31) and (32) into (33), the following is derived:


$$\begin{array}{c} \dot{S}=\dot{Z}+cZ\\ ={\dot{e}}_{\text{1}}+\xi {\dot{e}}_{2}+c(e_{1}+\xi e_{2})\\ ={\dot{\omega }}_{r}+\xi \dot{\beta }+c[(\omega_{r}-\omega_{d})+\xi (\beta -\beta_{d})] \end{array}$$ (34)

To reduce system chattering and improve response speed, an improved double-power reaching law is adopted, expressed as:


$$\dot{S}=-\varepsilon_{1}{\left|S\right|}^{\alpha }sgn(S)-\varepsilon_{2}{\left|S\right|}^{\tau }sgn(S)-q{\left|S\right|}^{\psi }$$ (35)

Where 0 < α < 1, τ>1, $\varepsilon_{1}$>0, $\varepsilon_{2}$ >0. By combining Equations (14), (34), and (35), the additional front-wheel steering angle *δ*_*f*_ of the active front steering (AFS) controller can be obtained as follows:


$$\begin{array}{c} \delta_{f}=\frac{\text{1}}{b_{1}+b_{2}\xi }[-\varepsilon_{1}{\left|S\right|}^{\alpha }sgn(S)-\varepsilon_{2}{\left|S\right|}^{\tau }sgn(S)-q{\left|S\right|}^{\psi }\\ \;-\beta (a_{11}+\xi a_{21}+\xi c)-\omega_{r}(a_{12}+\xi a_{22}+c)+c(\omega_{d}+\xi \beta_{d})] \end{array}$$ (36)

## 4. Simulation results and analysis

To verify the effectiveness of the coordinated control strategy of ASR and active front steering (AFS) designed in this paper, simulation analysis is conducted based on CarSim and Matlab/Simulink. The main technical parameters of the vehicle are shown in Table 1.

**Table 1:** Main technical parameters of the vehicle.

Name	Symbol	Value (Unit)
Vehicle mass	*m*	1412 kg
Tire radius	*r*	0.325 m
Wheel Rotational Inertia	*I*_*w*_	1.06 kg.·m^2^
Height of the center of mass	*h*_*g*_	0.54 m
Moment of inertia about the yaw axis	*I*_*z*_	1536.7 kg.·m^2^
Distance from front axle to center of mass	*L*_*f*_	1.015 m
Distance from rear axle to center of mass	*L*_*r*_	1.895 m
Distance from left wheel to center of mass	*B*_*l*_	0.8375 m
Distance from right wheel to center of mass	*B*_*r*_	0.8375 m
Front axle cornering stiffness	*k*_*f*_	−118610 N/rad
Rear axle cornering stiffness	*k*_*r*_	−94860 N/rad

### 4.1 Analysis of hill start performance

**Fig 9:**
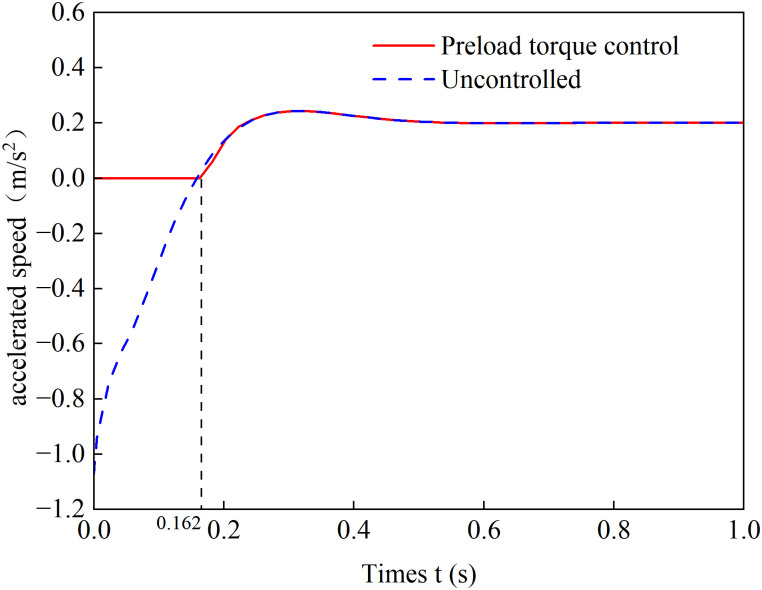
Slope starting acceleration curve.

To verify the effectiveness of the preload torque control strategy for hill starts, a simulation scenario was set up with a road slope *θ* = 0.1 and a road adhesion coefficient *μ* = 0.3. The vehicle began accelerating under two conditions: with and without preload torque control. The simulation results are shown in Fig 9.

Fig 9 presents the acceleration curves of the vehicle during hill start under both preload torque control and uncontrolled conditions. The results demonstrate that without preload torque control, due to the response delay of the in-wheel motor, the initial driving torque fails to overcome the gradient resistance, causing the vehicle acceleration to rapidly decrease to a negative value. A maximum rollback acceleration of −1.07 m/s² was observed, indicating significant vehicle rollback phenomenon. In comparison, with the preload torque control strategy implemented, the vehicle acceleration maintains at zero before 0.162 seconds. After this time point, the driving torque successfully overcomes the motion resistance, transforming the acceleration into positive values and enabling the vehicle to smoothly transition into the acceleration phase. These findings conclusively prove that the proposed preload torque control strategy effectively addresses the rollback issue during hill starts, ensuring stable and reliable vehicle launching performance.

### 4.2. Analysis of ASR performance

**Fig 10:**
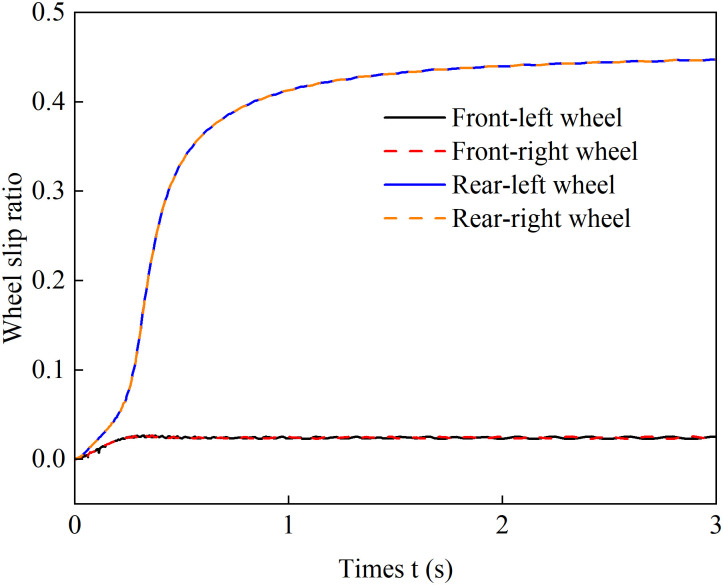
Evenly distributed control of wheel slip ratio under 1.5 m/s² acceleration.

**Fig 11:**
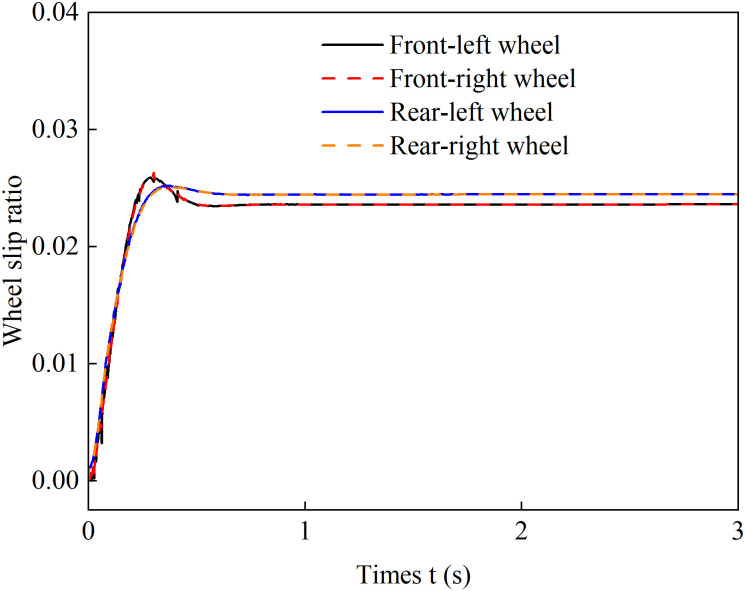
Coordinated control of wheel slip ratio under 1.5 m/s² acceleration.

To verify the effectiveness of this coordinated control strategy in actively suppressing wheel slip while balancing traction performance and road surface protection requirements, a full low-uniform-adhesion climbing scenario was set up with an adhesion coefficient *μ* = 0.3 and a slope *θ* = 0.1. The vehicle was configured to perform a starting acceleration climb with an acceleration of 1.5 m/s², and a comparative validation was conducted using a drive torque average distribution control method. The simulation results are shown in the following figure:

Figs 10 and 11 present the wheel slip ratio curves under average distribution control and coordinated control. A comparative analysis shows that with average torque distribution, the rear wheels exhibit severe slip, reaching a peak ratio of 0.45. In contrast, coordinated control maintains all wheel slip ratios at a low level, limiting the maximum value to 0.024, which confirms effective slip suppression. These results demonstrate that the proposed strategy dynamically adjusts the drive torque distribution among the wheels, effectively suppressing slip, improving adhesion utilization, and enhancing stability.

### 4.3. Analysis of straight-line climbing performance

To validate the effectiveness of the coordinated control strategy under uphill driving conditions, a straight driving road with a total length of 100 meters is selected. To simulate different slopes and varying road adhesion coefficients at the contact points of the four wheels when driving on compacted snow surfaces, the road conditions are designed as follows: From 0 to 30 meters, the road slope is 0, and the road adhesion coefficient is 0.3, representing a uniform low-adhesion surface. From 30 to 60 meters, the road slope is 0.06, and the road adhesion coefficients are 0.3 on the left side and 0.2 on the right side, representing a split-μ slope surface. From 60 to 100 meters, the road slope is 0.1, and the road adhesion coefficients are 0.3 on the left side and 0.2 on the right side, representing another split-μ slope surface. In the experiment, the vehicle was set to perform accelerated uphill motion with an initial speed of 20 km/h and an acceleration of 0.5 m/s². A comparative validation was conducted using three control strategies with good stability control effects: load distribution control, load distribution plus active front steering (AFS) control, and only optimization distribution control. The simulation results are shown in the following figure.

**Fig 12:**
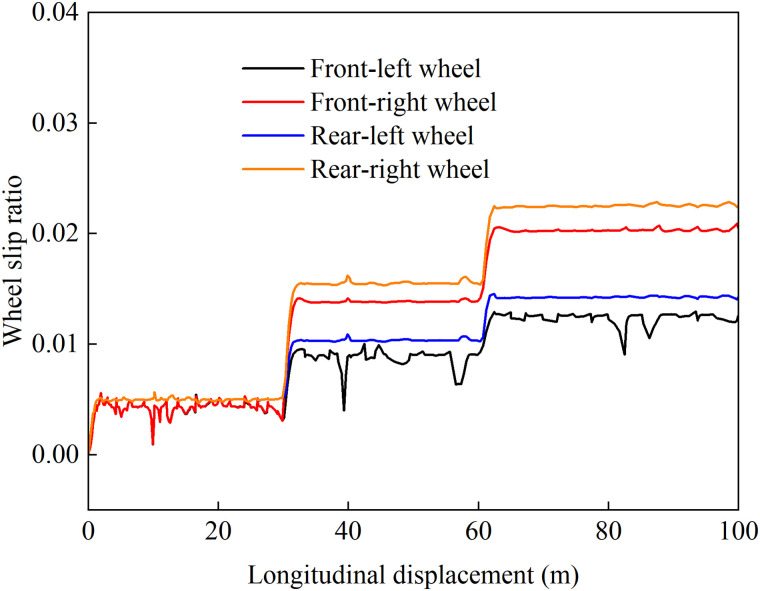
Wheel slip ratio under load distribution control during uphill acceleration.

**Fig 13:**
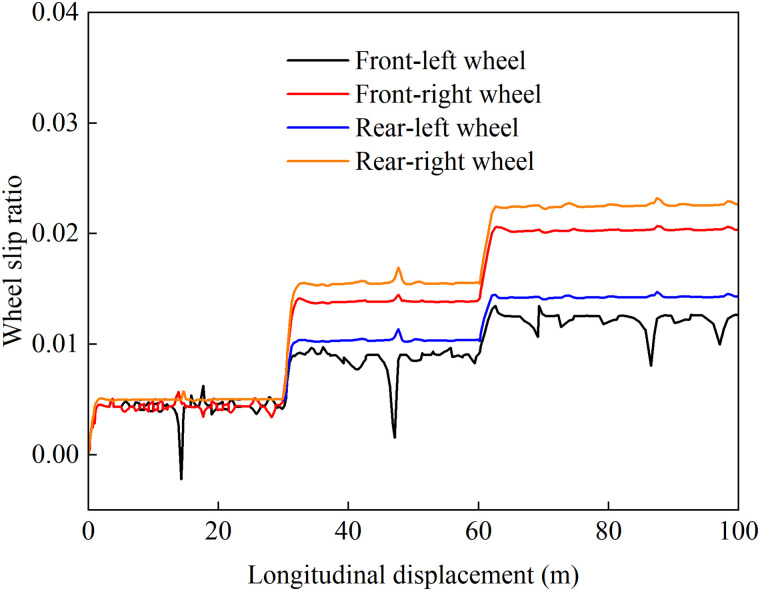
Wheel slip ratio under load distribution and active front steering control during uphill acceleration.

**Fig 14:**
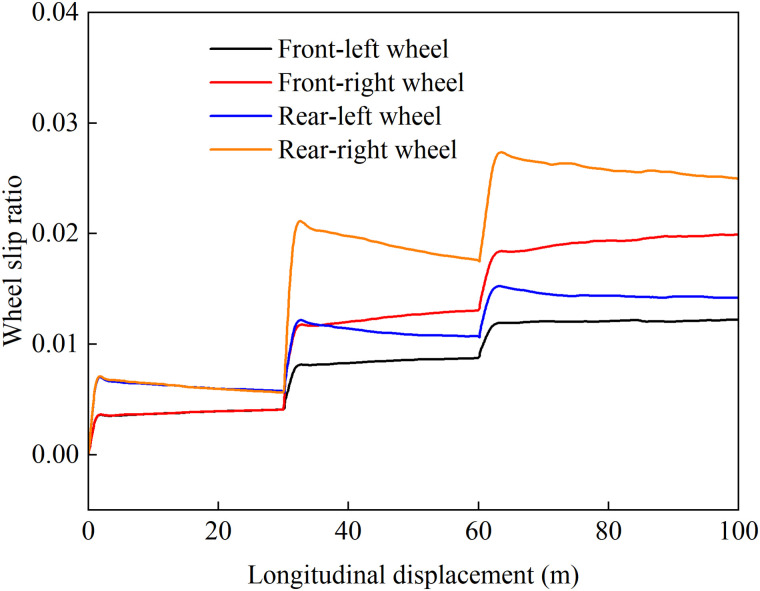
Wheel slip ratio under only optimization distribution control during uphill acceleration.

**Fig 15:**
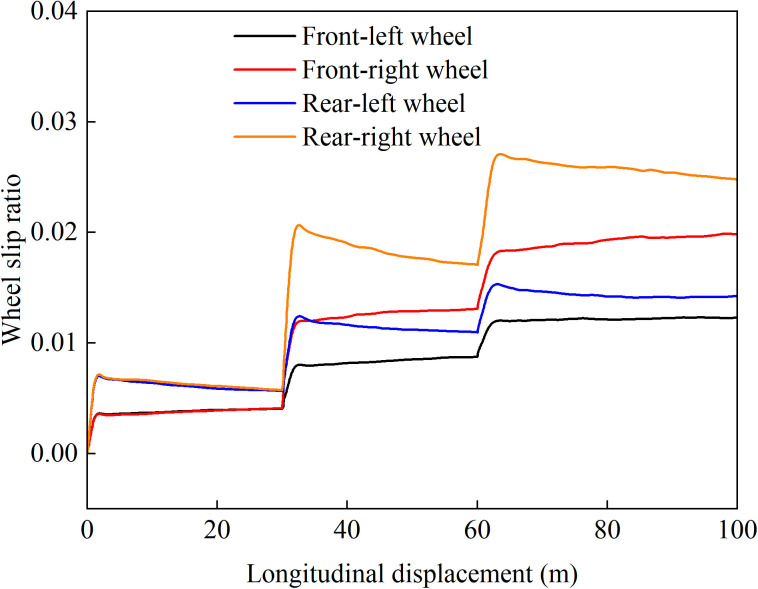
Wheel slip ratio under coordinated control during uphill acceleration.

Figs 12–15 show the curves of wheel slip ratio versus longitudinal vehicle displacement under four different control strategies. The results indicate that under load distribution control and load distribution plus AFS combined control, the slip ratios of all four wheels—especially the left front wheel—exhibit significant high-frequency oscillations, indicating frequent fluctuations in wheel driving force, which severely degrades the vehicle’s ride quality. In contrast, the optimized distribution and coordinated control strategies achieve multi-objective coordinated optimization in distributing drive torque to all four wheels, maintaining the slip ratios within a low range without noticeable oscillations. This demonstrates smoother vehicle operation, optimized utilization of available adhesive friction, and avoidance of destructive snow-plowing effects, thereby significantly enhancing the vehicle’s trafficability.

**Fig 16:**
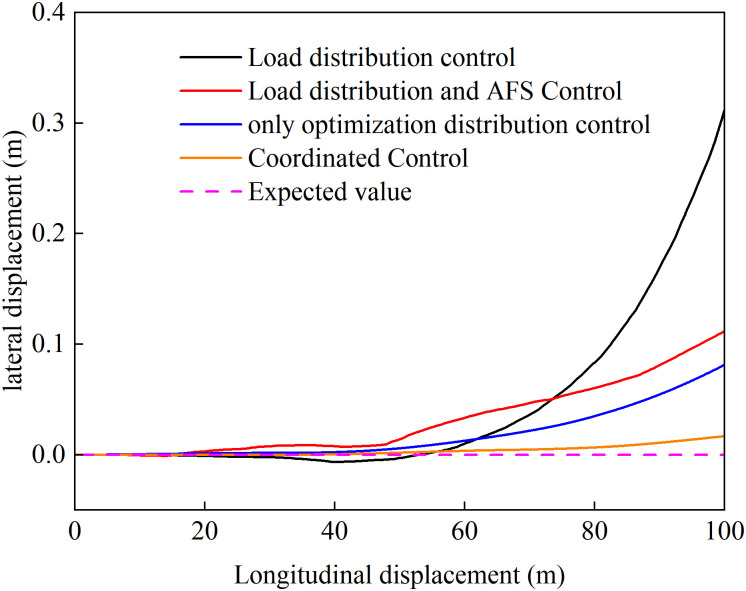
Vehicle trajectory during uphill acceleration.

Fig 16 shows the vehicle trajectories under four different control strategies. Comparative analysis reveals that when the load distribution control strategy is adopted, the vehicle trajectory exhibits significant deviation with a maximum offset of 0.31 m. The root cause analysis indicates: the continuous accelerated uphill motion generates high demand for driving torque, while the low-adhesion and left-right asymmetric road conditions result in different longitudinal driving forces on both sides under identical driving torque. This driving force difference creates undesired yaw moment, triggering vehicle attitude deflection. Simultaneously, factors like acceleration, uphill climbing, and road excitation induce dynamic vertical load transfer on wheels. The load distribution strategy adjusts torque allocation based on real-time loads, under such extreme conditions characterized by high demand, low adhesion, and asymmetry, this strategy merely passively responds to load variations without dynamic adjustment of wheel driving torque. Consequently, it easily falls into an oscillatory cycle of “slip → load transfer → torque redistribution → aggravated slip,” ultimately causing severe deviation from the intended path. For the vehicle employing load distribution plus AFS, although the trajectory deviation is reduced, relying solely on AFS cannot fully eliminate the effects of undesired yaw moment. In contrast, vehicles using optimized distribution and coordinated control strategies demonstrate trajectories almost identical to the ideal path. The results demonstrate that this strategy achieves coordinated optimization of slip ratio control and yaw stability to dynamically adjust driving torque distribution in real time, effectively constraining excessive wheel slip on either side while suppressing undesired yaw moment, thereby significantly enhancing vehicle driving stability.

**Fig 17:**
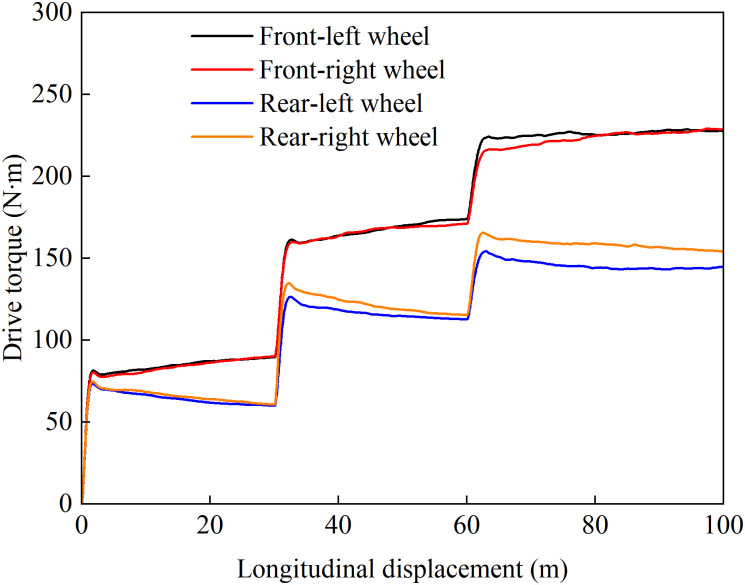
Wheel torque output during uphill acceleration under coordinated control.

Fig 17 presents the driving torque distribution curves of individual wheels versus vehicle longitudinal displacement under the coordinated control strategy. As shown in the figure, the proposed strategy can effectively adapt to varying road conditions. In the 0-30m section (uniform low-adhesion surface with 0% slope), the torque distribution among the four wheels generally follows the load proportion while maintaining left-right symmetry, ensuring straight-line stability. As the slope increases, the total required driving torque rises significantly, leading to corresponding torque increments at each wheel. When the traction force of the high-adhesion-side wheels approaches saturation, the strategy proactively allocates more driving torque to the low-adhesion-side wheels to maintain the slip ratio within a safe range. This approach ensures both ASR performance and directional stability while preserving driving dynamics. The results demonstrate that the proposed coordinated control strategy effectively suppresses excessive wheel slip and avoids drastic torque fluctuations caused by passive responses to rapid load transfer or slip feedback, significantly improving ride quality.

### 4.4. Analysis of steering performance

To verify the effectiveness of the coordinated control strategy under steering conditions and demonstrate its capability in balancing dynamics and stability during accelerated turning, a simulation was conducted on a uniform low-adhesion road surface with a friction coefficient of 0.3. Apply a sinusoidal steering wheel input with an amplitude of 45° and a period of 4s at the 3rd second to simulate cornering maneuver conditions. The vehicle performed an accelerated turning motion with an initial speed of 20 km/h and an acceleration of 0.5 m/s². Comparative validation was carried out using load distribution control, load distribution plus active front steering (AFS) control, and only optimization distribution control. The simulation results are shown in the following figure.

**Fig 18:**
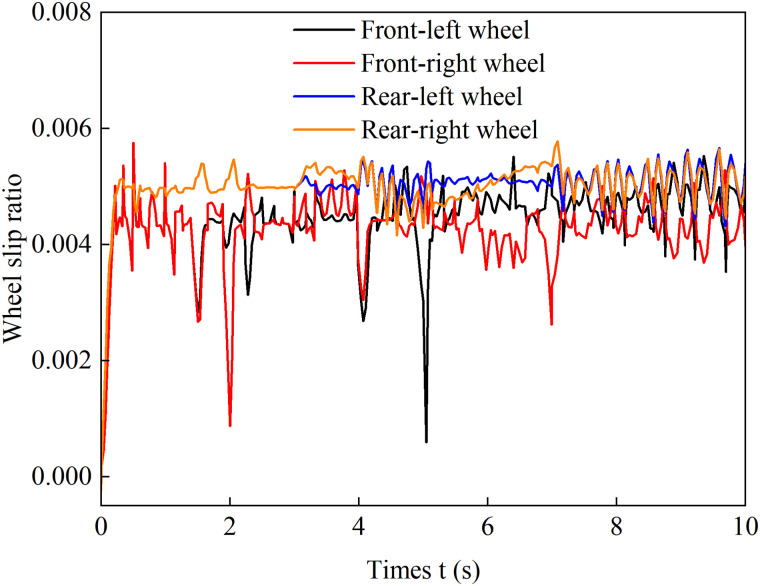
Wheel slip ratio under load distribution control during accelerated turning.

**Fig 19:**
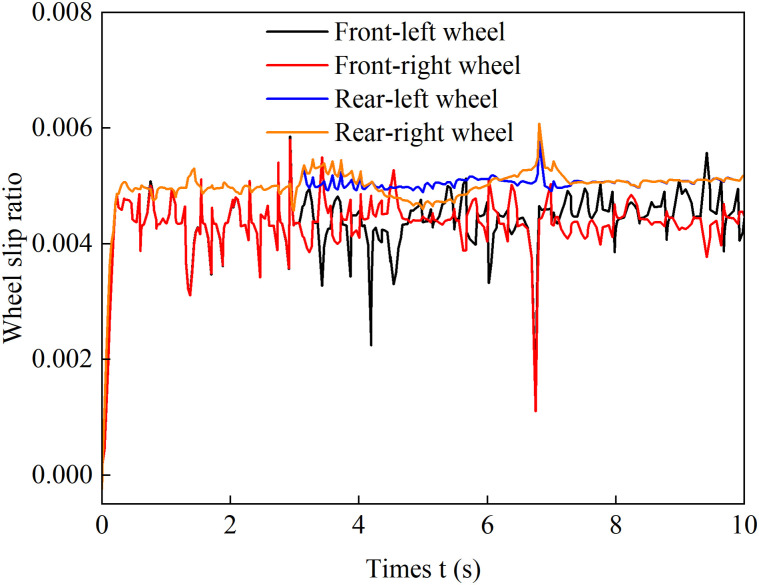
Wheel slip ratio under load distribution and active front steering control during accelerated turning.

**Fig 20:**
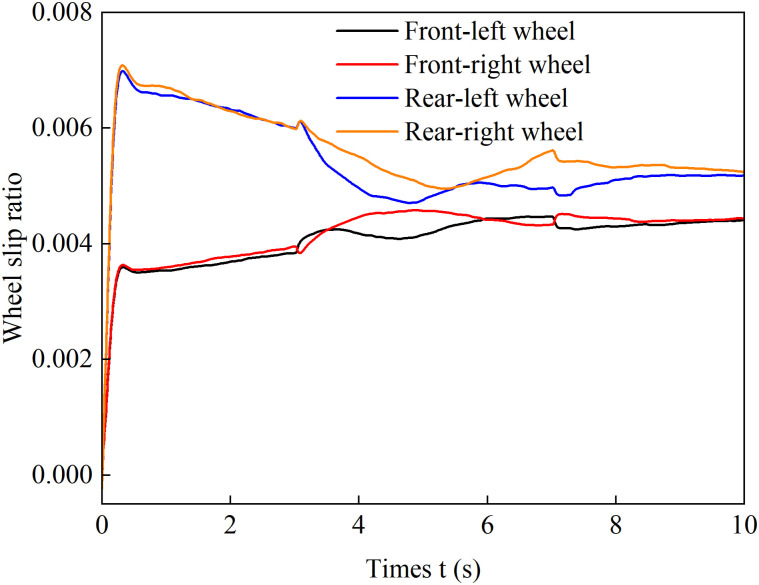
Wheel slip ratio under only optimization distribution control during accelerated turning.

**Fig 21:**
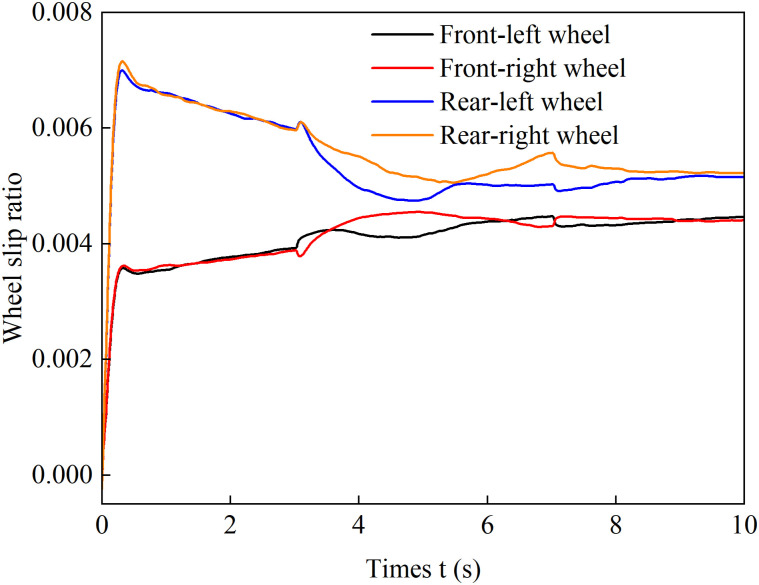
Wheel slip ratio under coordinated control during accelerated turning.

Figs 18–21 show the wheel slip ratio curves over time under four different control strategies. Under both the load distribution control strategy and the load distribution + AFS combined control strategy, the wheel slip ratios exhibit significant oscillations, indicating that undesired yaw moments are generated, leading to vehicle instability. In contrast, under the optimized distribution control strategy, all wheel slip ratios remain at relatively low levels, with no noticeable vibrations during steering maneuvers, and the variation process is much smoother. These results demonstrate that the optimized distribution strategy effectively balances the driving forces between the left and right wheels, suppresses vehicle sideslip, and enhances both longitudinal and lateral stability.

**Fig 22:**
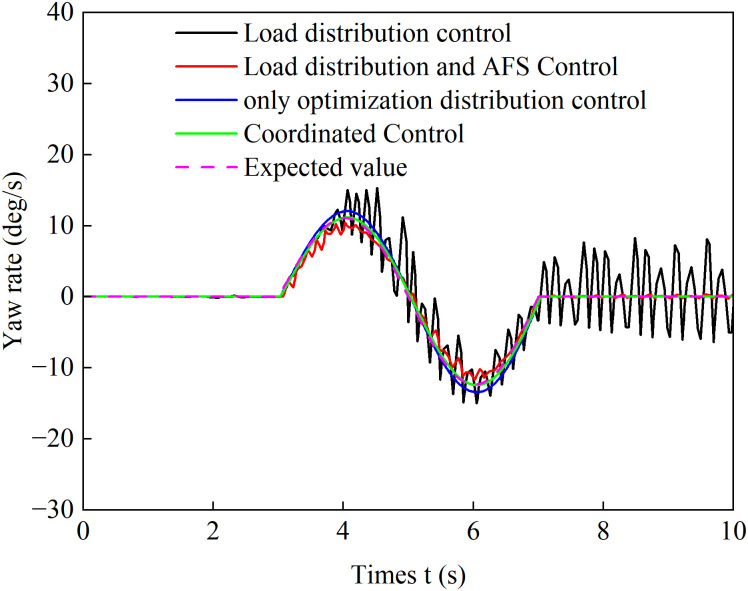
Yaw rate comparison during accelerated turning.

**Fig 23:**
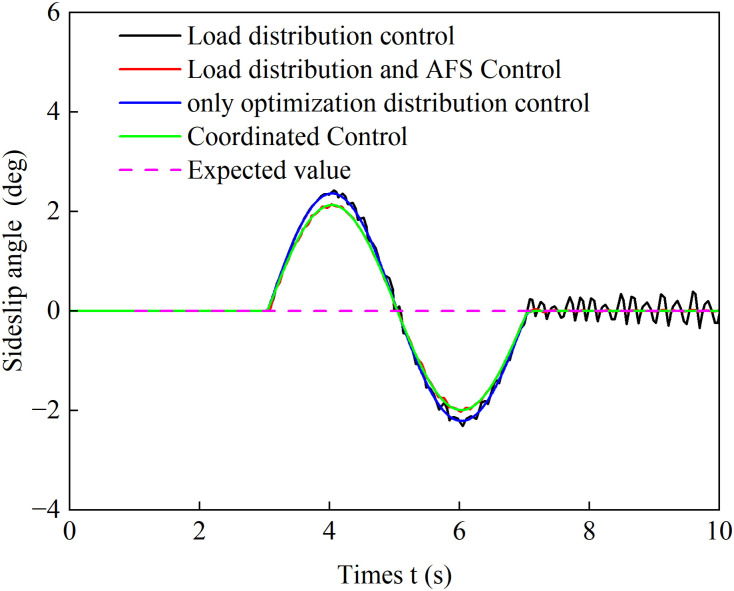
Sideslip angle comparison during accelerated turning.

**Fig 24:**
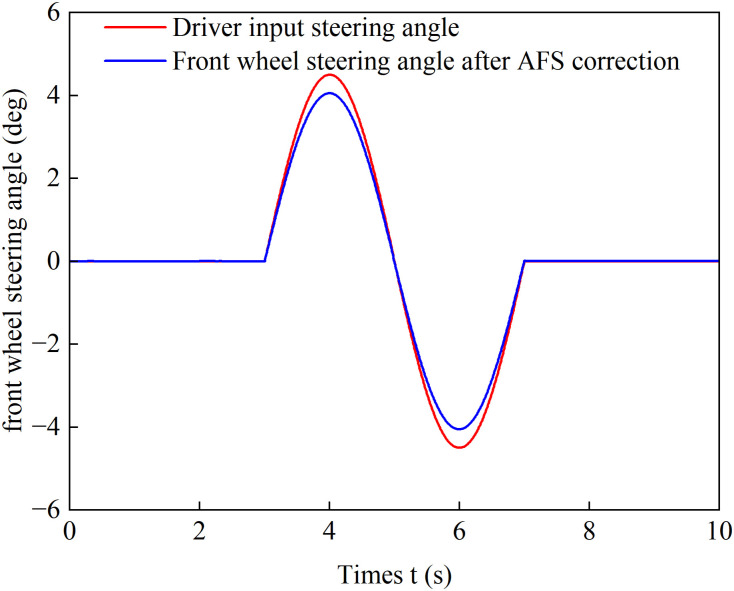
Front-wheel steering angle under coordinated control during accelerated turning.

Figs 22 and 23 show the yaw rate and sideslip angle curves over time during accelerated turning under the four different control strategies, respectively. Fig 24 displays the front-wheel steering angle under the coordinated control strategy during accelerated turning. Analysis of Figs 22 and 23 shows that under the load distribution control, the vehicle’s yaw rate and sideslip angle exhibit significant oscillations with large deviations from the ideal values, indicating poor vehicle stability. Under the combined action of load distribution + AFS, compared with pure load distribution control, the oscillation amplitudes of the vehicle’s yaw rate and sideslip angle are reduced. However, combined with the observed wheel slip rate oscillations in Figs 18 and 19, it indicates that although this strategy introduces AFS for front-wheel steering angle compensation, it fails to effectively suppress vehicle instability caused by improper drive torque distribution. Under the only optimized distribution control, the vehicle’s yaw rate and sideslip angle show no significant oscillations, indicating that the torque distribution strategy based on multi-objective optimization effectively balances the torque demands of each wheel during accelerated turning, suppresses vehicle oscillations, and improves driving stability. On the basis of suppressing chattering with a only optimization allocation strategy, coordinated control further brings the vehicle’s yaw rate and sideslip angle closer to the ideal values. This indicates that the coordinated control of torque optimization allocation and active front steering (AFS) more accurately tracks the desired yaw rate and sideslip angle, thereby achieving a higher level of directional stability during accelerated turning maneuvers.

**Fig 25:**
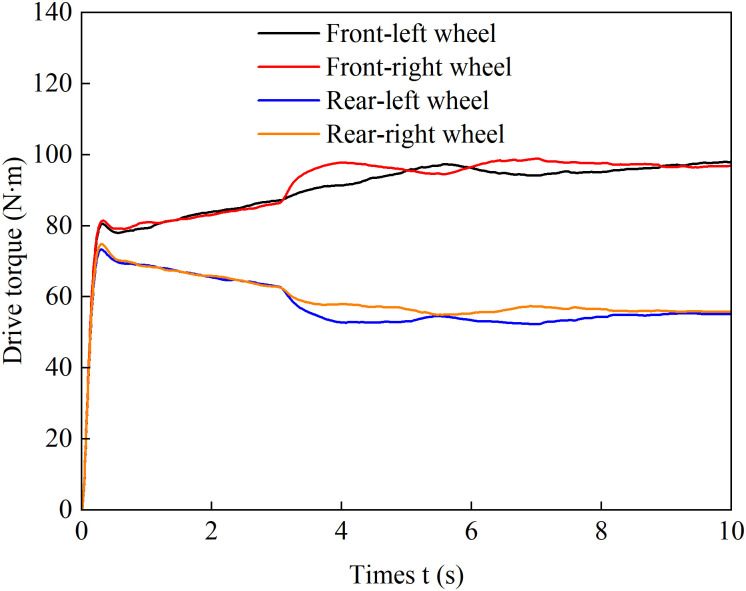
Wheel torque output under coordinated control during accelerated turning.

Fig 25 shows the driving torque output of each wheel during accelerated turning maneuvers under coordinated control. As can be seen from the figure, the coordinated control can effectively respond to changes in vehicle states under different steering conditions. When the vehicle is making a left turn (3–5 seconds), the right wheel has a larger turning radius and requires a higher linear velocity. The coordinated control increases the torque on the right wheel to generate the necessary yaw moment for steering assistance. As the steering angle gradually decreases, the turning radii of both wheels progressively reduce, and the torque difference between the two drive wheels also diminishes accordingly. During a right turn (5–7 s), the left wheel has a larger turning radius. From 5–6 s, the coordinated control continuously increases the left wheel’s torque while reducing the right wheel’s torque. From 6–7 s, as the difference in turning radii between the two wheels gradually decreases, the left wheel speed becomes higher. At this point, the coordinated control begins to increase the right wheel’s torque to meet the Ackermann steering speed difference requirement. During straight-line driving (after 7 s), the torque difference rapidly converges to suppress undesired speed differences between the wheels, minimize slip rates, and maintain straight-line driving stability. In summary, this coordinated controller dynamically adjusts the torque distribution among the drive wheels. During acceleration and steering maneuvers, it actively generates the required wheel speed difference, while during straight-line driving, it suppresses undesired speed discrepancies. By globally balancing the vehicle’s comprehensive demands for anti-slip performance, stability, and drivability, it significantly enhances the overall driving performance.

## 5. Conclusion

In-wheel motor driven electric vehicles face challenges of wheel slip and instability under low-adhesion conditions such as compacted snow. Existing solutions present certain limitations: conventional optimal slip ratio tracking methods excessively sacrifice lateral tire force reserve while pursuing maximum longitudinal force, adversely affecting lateral stability, while most current longitudinal-lateral coordination control strategies neglect active wheel slip ratio constraints, failing to fundamentally prevent adhesion deterioration caused by wheel spin. To address these issues, this study proposes a coordinated control strategy integrating drive anti-slip control and active front steering based on dynamic slip ratio constraints. The main work and conclusions are as follows:

(1) A dynamic slip ratio constraint method was developed to actively maintain the slip ratio within a safe range, effectively suppressing wheel slip while preserving lateral tire force capacity and ensuring driving safety.
(2) A feedforward-feedback composite control architecture was designed to achieve deep coordination between anti-slip control and active steering. The feedforward module distributes drive torque via a multi-objective optimization algorithm to suppress undesired yaw moments, while the feedback module generates front-wheel compensation angles using a sliding mode control algorithm for precise compensation. This architecture avoids disturbances caused by control authority switching in conventional methods and enables seamless longitudinal-lateral stability integration.
(3) Co-simulation using CarSim and MATLAB/Simulink demonstrated the effectiveness of the proposed strategy. Results show significant suppression of excessive wheel slip, with yaw rate and sideslip angle smoothly and steadily tracking their reference values. The strategy enhances anti-slip performance and directional stability on icy/snowy surfaces while balancing driving efficiency and road surface protection, offering an effective solution for active safety control of in-wheel motor driven electric vehicles in winter conditions.

Limitations and Future Work: This study idealizes steering actuation dynamics, assuming perfect execution of the AFS compensation angle, which may lead to over-optimistic performance evaluation under high-frequency or extreme conditions. Future work will develop higher-fidelity steering system models incorporating response delays, rate saturation, and nonlinearities, and will explore feedforward compensation or inverse model-based strategies to mitigate the impact of actuator dynamics.
